# Acute Blood Loss Anemia in the Setting of Abdominal Aortic Aneurysm Rupture in a Jehovah's Witness

**DOI:** 10.7759/cureus.11400

**Published:** 2020-11-09

**Authors:** Bolajoko O Fayoda, Dhruv Patel, Michele Davis, Obed Adarkwah, Jose Orsini

**Affiliations:** 1 Internal Medicine, The Brooklyn Hospital Center, Brooklyn, USA; 2 Pulmonary and Critical Care Medicine, The Brooklyn Hospital Center, Brooklyn, USA; 3 Critical Care Medicine, The Brooklyn Hospital Center, Brooklyn, USA

**Keywords:** critical anemia, acute blood loss anemia, abdominal aortic aneurysms, surgical critical, jehovah’s witnesses, erythropoetin, endovascular aneurysm repair, bloodless approach, critical hemorrhagic shock

## Abstract

Acute blood loss anemia occurs due to many variants. The standard of care in managing acute blood loss anemia is challenged in this case. Jehovah's Witnesses's (JW) management of blood loss anemia continues to remain a controversy in medicine since they do not allow the use of blood products. This case highlights the management of acute blood loss anemia, utilizing a multidisciplinary bloodless approach in a JW who underwent an endovascular aneurysmal repair (EVAR) of an impending rupture of abdominal aortic aneurysm (AAA). The severity of anemia with hemoglobin of 2.7 g/dL and survival outcome is unique; however, the minimal hemoglobin level required to sustain life is still unclear.

## Introduction

Jehovah’s Witnesses (JW) does not allow the transfusion of allogeneic blood products. As this religious community is becoming a large part of our patient population, we must understand ways in providing therapeutic approaches while honoring their religious beliefs. This case remains interesting as the severity of anemia warranted acute aggressive medical-surgical interventions. Often, healthcare providers are presented with cases like ours, which poses a challenge to our various skillsets and may lead to a medico-legal arena. Although JW patients decline blood transfusion, however, it should not be considered as a refusal of medical treatment. Bloodless medicine and surgery (BMS) have been recognized as a therapeutic modality for people who cannot receive a blood transfusion. This involves the use of peculiar practices along with a multidisciplinary effort, thus allowing for a calculated approach in reducing morbidity and mortality in these patients.

## Case presentation

**Figure 1:**
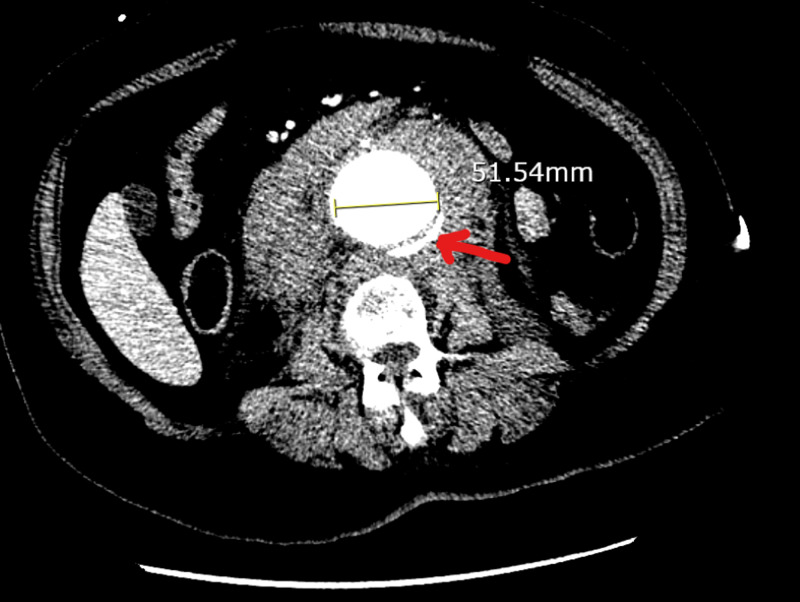
A CTA abdomen and pelvis showing a 5.1 cm AAA with a high-attenuating crescent sign (red arrow) CTA: computed tomography angiogram; AAA: abdominal aortic aneurysm

This case describes a 55-year-old African American female with a known medical history of hypertension, obesity, Marfan syndrome, and abdominal aortic aneurysm (AAA). She complained of severe low back pain after lifting weights at the gym several hours prior to admission. The patient described her pain as constant, stabbing, 9/10, positional with radiation to the lower abdomen, with no alleviating or aggravating factors. On presentation, vital signs were stable and physical examination was unremarkable. Due to her history and lack of appreciable indications from the physical examination, a computed tomography angiography (CTA) of the abdomen and pelvis was performed demonstrating a 5.1 cm in diameter abdominal aortic aneurysm, originating below the level of the renal arteries with an identifiable crescent sign concerning for a rupture (Figure 1).

**Figure 2:**
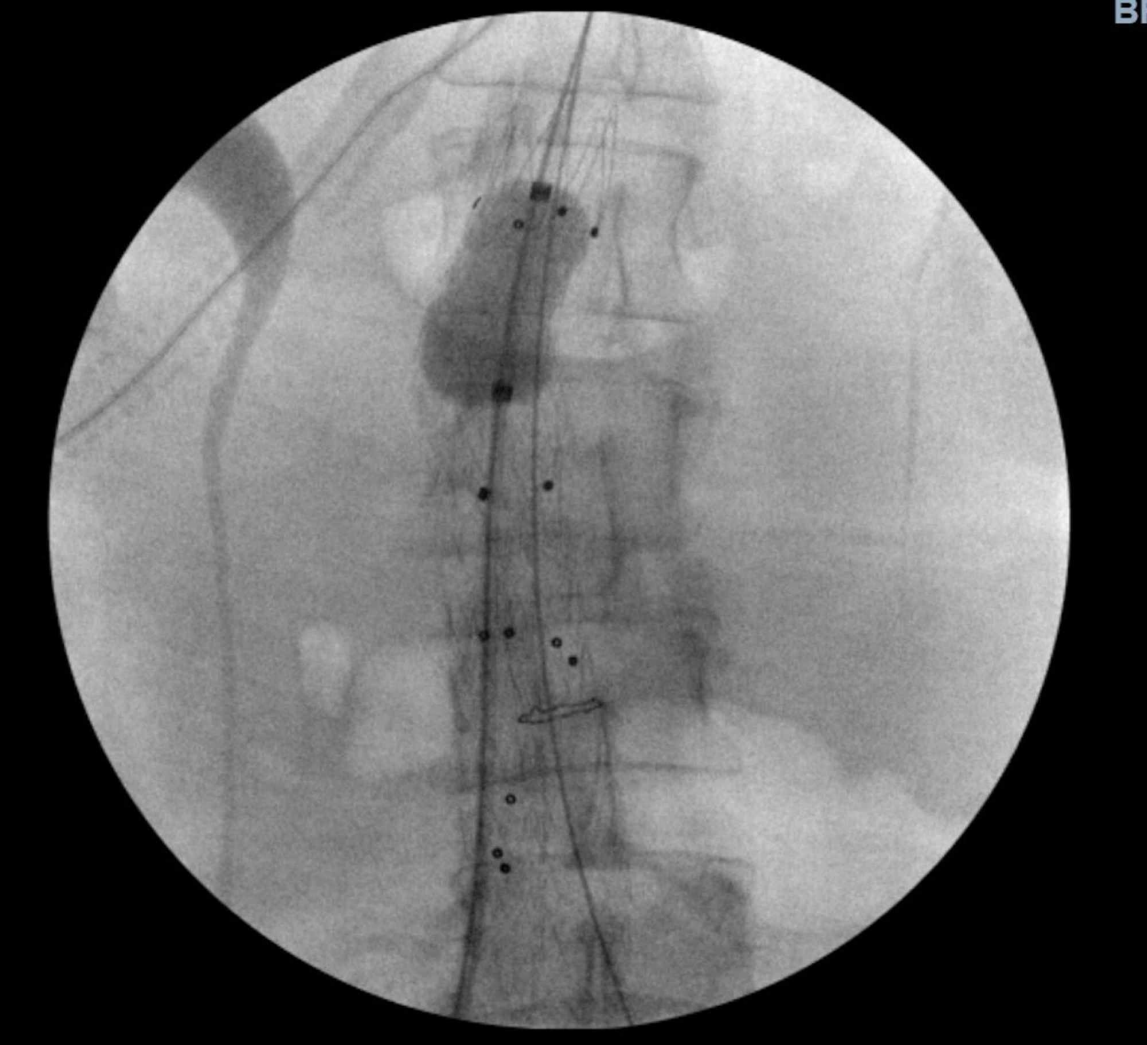
Fluoroscopic view stent placement of EVAR EVAR: endovascular aneurysmal repair

Complete metabolic panel (CMP) and complete blood count (CBC) were unremarkable, including an initial hemoglobin (Hgb) level of 11.7 g/dL. Coagulation studies were also within normal limits. This hospital course was complicated by hemodynamic instability with a systolic blood pressure of 70 mmHg, and acute changes in mentation. The emergency room team initiated aggressive intravenous fluids resuscitation with crystalloids, intubation, initiation of mechanical ventilation, and maintaining permissive hypotension with mean arterial pressure (MAP) goal of 50-65 mmHg. At this point, the patient was immediately taken to the operating room by the vascular surgery team and underwent a successful endovascular aneurysm repair (EVAR) (Figure 2). She remained intubated and post-procedure developed hemorrhagic shock in the setting of possible ruptured AAA. The patient remained hemodynamically unstable, critically ill, and was transferred to the ICU, and initiated on vasopressors (norepinephrine, vasopressin, and epinephrine). Post-procedure hemoglobin/hematocrit (H/H) level was 3.9/12 g/dL, which then trended down to 2.7/8 g/dL (normal range 11.5-14.1/ 35-42 g/dL). Because of this patient’s religious beliefs, bloodless approach management was initiated (non-blood products). The patient was started on hydroxyethyl starch (HES), erythropoietin (EPO), intravenous iron sucrose, intravenous folate (B9), intravenous thiamine (B1), intravenous cyanocobalamin (B12), and vitamin C therapy. On day 7 of ICU admission, the patient was weaned off vasopressors, and repeat H/H was 5.8/17. On day 10 of ICU admission, the patient was successfully extubated, and follow up H/H levels were 5.3/17. On day 15 of ICU admission, she was transferred to the medicine surgery ward, and H/H levels improved to an impressive 8.7/27 g/dL. On day 18 of hospital admission, the patient was sent to acute rehabilitation for 14 days and discharged home with H/H levels of 9.5/31 g/dL. 

## Discussion

Jehovah's Witnesses (JW) are a vastly growing community worldwide. Not understanding how to manage severe acute blood loss anemia urgently in this population group will pose a threat to the lives we have taken an oath to protect. Although JW patients decline blood transfusion, however, it should not be considered as a refusal of medical treatment. Usually, a typical bloodless treatment approach is a peri-operative planned occurrence. Although we were faced with hard choices in the care of this patient, seeking alternatives in the management of acute blood loss anemia became our quest for a successful outcome. The Witness's refusal of blood transfusion brings forth challenges to the current standard of care, and this belief does not only restrict current management but also demands a new approach in the care of blood loss anemia. This approach renders a goal, and it is to pursue a constructive multidisciplinary effort to achieve a strategic gain that will most likely grant a favorable outcome.

It is important to note the strong correlation between severe anemia and poor patient outcomes, particularly when there are comorbidities or multiple chronic diseases. There are variants to acute blood loss anemia; however, knowing how patients respond to decreased H/H levels, and understanding these individual responses is important as it is essential to how they harness physiological responses for survival benefit. The hemoglobin level (2.7 g/dL) post-operatively was concerning, and our patient remained in a critical condition. The discussion of survival without blood transfusion was had with family as well as the healthcare proxy, and it was expressed that not receiving blood products is essential to the core value of their faith. This only reassured one thing; that our life-saving strategy would prove difficult but achieving the ultimate goal requires an all-hands-on-deck approach. A retrospective study of JWs' who declined blood products showed that mortality increased progressively as hemoglobin decreased below 6 g/dL, with >50% mortality amongst patients with the lowest postoperative hemoglobin of 2.1 - 3.0 g/dL, and 100% mortality if 1.1 - 2.0 g/dL [1]. This evidence of patients declining blood transfusion shows a decrease in survival with decreasing levels of hemoglobin concentration; however, the minimal hemoglobin level required to sustain life remains unclear [1-3].

The critical hemoglobin can vary from patient to patient which is supported by several factors such as the level of oxygen required, adaptive responses, and the reserve capacity in various organs at the cellular level [3]. At this time, our patient remains on mechanical ventilation, and we feared the odds of survival were against our patient. At this juncture, a sorely needed comprehensive management approach would likely yield a favorable outcome despite limited literature or data. There are reports of a comprehensive set of strategies developed to meet the specific needs of JWs' and these strategies are collectively called “bloodless medicine and surgery” (BMS) [3]. This strategy allows for a set protocol involving a multidisciplinary effort to facilitate optimizing Witness's or patients who do not want a blood transfusion. There is no standard BMS protocol and can vary from institutions to institutions. The role of mechanical ventilation in severe anemia is documented and poses a threat to patients to be weaned off the device. In a study of anemic patients with known chronic obstructive lung disease, with a mean hemoglobin, 8.7 g/dL, and who were unable to be liberated from mechanical ventilation, all were successfully extubated within four days of being transfused to a mean hemoglobin level of 12.4 g/dL [4-5]. At hemoglobin of 2.7 g/dL, our patient remains hemodynamically unstable and requires measures of resuscitation. Although our patient did not have a history of lung disease however, she was extubated at a hemoglobin of 5.3 g/dL. The critical level of anemia to sustain basic physiologic standards may be unknown; however, there is evidence and data to support that most organs perform poorly if the level of critical anemia is accompanied by hypovolemia. HES provides a high colloid osmotic pressure to assist in maintaining circulatory volume by increasing the viscosity effect as it assists in maintaining oxygen delivery [3]. This was essential to provide sufficient circulating volume to preserve perfusion. HES has been shown to restore circulatory volume in shock models [3]. Thus, facilitating volume expansion in our critical patient maintained circulatory volume and provided perfusion to vital organs. However, there are reports of HES facilitating increase bleeding effect thus, it remains controversial the negative impact on the coagulation profile [3,6-7].

There are alternatives to improve red blood cell production without blood transfusion. Blood is an extremely complex fluid and comprises red cells, white cells, platelets, and plasma. Moreover, the efficiency of a red blood cell function is dependent on the blood transport of oxygen (O2) and carbon dioxide (CO2). This is a key physiological process that is dependent on the O2, CO2, and H+ binding properties of hemoglobin. The binding properties are facilitated by the enzyme carbonic anhydrase, red blood cell-specific membrane, cytoplasmic proteins by the red blood cell environment [4]. Iron is found predominantly in hemoglobin and stored in the body as ferritin. Iron maintains a significant role in the body as an oxygen-carrying molecule in heme; as iron irreversibly binds oxygen. A randomized clinical trial of 200 patients in the surgical intensive care unit who received enteral ferrous sulfate vs placebo, showed these patients were not likely to receive a transfusion of blood product. Also, no statistical significance of infection rates was identified, and intravenous iron therapy had better efficacy over enteral administration due to blockade via intestinal absorption by hepcidin [4,8]. However, it will be beneficial to advocate for further substantial clinical trials before iron can be officially recommended for optimal use in critically ill anemic patients. 

Erythrocytes (red blood cells) are the most commonly formed elements that carry oxygen into the cells via hemoglobin. Erythropoiesis occurs via erythropoietin (EPO), a major hormone that stimulates erythrocytes. EPO is a peptide produced through the renal system and regulates erythrocyte production via a collaborative feedback system. Our patient received a high dose of EPO (20,000 units) for a total course of 18 days. Though studies reflect a response to hemoglobin with the use of EPO in a meta‐analysis of nine randomized‐controlled trials studying epoetin alfa in critically ill medical and surgical patients with a reduction of H/H levels in the likelihood of receiving a transfusion. However, no effect on mortality was observed [2,9]. Lack of EPO is noted in critically ill patients, and some studies show a fixed dose of erythropoietin administered weight-based in units/kg and varied between 36,750 to 160,000 units weekly. Additionally, recombinant erythropoietin has been used to stimulate erythropoiesis to mitigate anemia, and reduce the need for blood transfusions [9]. The essential factors of erythropoiesis include iron, folate (B9), cyanocobalamin (B12), and zinc under the influence of EPO, thyroxine, androgens, cortisol, and catecholamines [4]. Moreover, the use of these nutritional supplements may not be a routine standard of care however, promoting red blood cell proliferation, hemoglobin synthesis, and hematopoietic precursor maturation was vital to our cause.

While JWs refuse transfusion of blood products, the importance of having surgical procedures is not novel to the community despite its risks. Our patient experienced a shear force on a vessel creating a bleeding point requiring emergent intervention. Most situations for JWs requires peri-procedural planning, obtaining consents as well as imparting education regarding the procedure(s) in order to guide this patient population and their families. However, in our case, we explained complications as well as risks versus the benefit of EVAR with the likelihood of requiring an open repair procedure but we lacked time to prepare, as source control was necessary. The evidence of hyperattenuating crescent sign on imaging is a sign of impending rupture; hyperattenuating crescents have been attributed histopathologically to hemorrhage into the mural thrombus or the aneurysm wall, with clefts of blood seeping from the lumen into the thrombus. The hemorrhage later penetrates the aneurysm wall, which weakens the wall. This places the aneurysm at risk for frank rupture, and prompt surgical evaluation is sorely needed, as an intervention is required [10]. Also, it is important to note that source control of bleeding from EVAR allowed for the achievement of hemostasis. This was a bloodless surgery; as part of a multidisciplinary effort to achieve the best outcome for our patient. 

Given the nature of our patient, keeping further blood loss to a minimum as part of the operative skill was a necessity. Bloodless treatment for JWs in this setting required the ultimate multidisciplinary approach (medicine, surgery, anesthesia, pharmacokinetics). This includes performing phlebotomy at a minimum while monitoring levels of hemoglobin. However, point-of-care arterial blood gas would be necessary to manipulate and maintain oxygenation while ventilated. [3]. Most of the specimen tubes to obtain labs vary from facility to facility however, in this patient we relied on our pediatric tubing to obtain minimal blood samples to optimize care by grouping laboratory specimens. The pediatric tube requires a minimum of 2 mL and can be as low as 0.5 mL of blood, and this can reduce blood loss from a range of 33 - 80% [4]. Other forms of blood loss were avoided, such as wasting blood from the arterial line or central venous catheters, as it provides ease of phlebotomy. While limited literature exists on a conservative strategy to minimize blood loss, however, there is a need for more studies or initiatives to be implemented. 

JWs will continue to grow and become a greater part of our community. The greatest decision not to receive blood transfusion by this community falls on scripture(s) within the Bible to protect their faith, and is an oath they have sworn to live by. We as professionals took an oath to protect all lives from harm, and that we have sworn to live by as well. Therefore, we must challenge ourselves and the standard of care to lift the barricades of limitations in providing current adequate care to JWs with blood loss anemia. There are risks to receiving allogeneic blood transfusions, this thought has gained acceptance widely. The deleterious effects of blood transfusion are apparent, such as acute lung injury, transfusion-related acute lung injury, transfusion-associated circulatory overload, and transfusion-related immunomodulation [4]. This potentially can also lead to increased risks of nosocomial infection, such as; human immunodeficiency virus (HIV), hepatitis B, C, and others on the list of potentially transmissible diseases [4,11]. Furthermore, bloodless surgery is part of a coordinated multidisciplinary approach, as it combines various skillset to facilitate blood conservation strategies [11]. While this effort is not novel, we nevertheless recommend that various institutions embrace a standard protocol, and facilitate bloodless enrichment programs.

## Conclusions

Jehovah's Witnesses are willed to a certain life choice. The degree to which our patient survived was in her will to recover. It was a clear case of perseverance, persistence, and resilience despite all obstacles. The survival outcome from the level of critical anemia is unique. This case is pertinent due to our patient’s particular religious beliefs trammeling the standard of care. While JWs go through peri-operative planning to maximize success with interventions however, this case did not offer much time to plan. Furthermore, obtaining source control remains a primary approach in managing acute blood loss in JWs' with minimal blood loss. Although JWs' patients decline blood transfusion, it should not be considered as a refusal of medical treatment. Likewise, the use of non-blood products as well as nutritional supplements can provide favorable results as seen in our patient, and healthcare providers need to be familiar with alternatives. The BMS is a multidisciplinary effort involving various specialties which allows for a calculated approach in reducing these patient's morbidity and mortality. There is limited literature as well as data in response to the optimal management of a JW patient, as they do not allow transfusion of blood products. Now, while the minimal hemoglobin level required to sustain life is still unclear, further randomized trials may help provide clarity. 
